# 2024 Global Symposium on Health Systems Research

**DOI:** 10.2471/BLT.24.291344

**Published:** 2024-03-01

**Authors:** Hirotsugu Aiga, Ikuo Takizawa, Stephanie M Topp, Hitoshi Murakami, Sarah Louise Barber, Yasuhiko Kamiya, Kumanan Rasanathan, Adnan A Hyder

**Affiliations:** a School of Tropical Medicine and Global Health, Nagasaki University, 1-12-4 Sakamoto, Nagasaki, 852-8523, Japan.; b JICA Ogata Sadako Research Institute for Peace and Development, Japan International Cooperation Agency, Tokyo, Japan.; c College of Public Health, James Cook University, Townsville, Australia.; d Department of Human Resource Development, National Center for Global Health and Medicine, Tokyo, Japan.; e World Health Organization Centre for Health Development, Kobe, Japan.; f Japan Association for Global Health, Bureau of International Health Cooperation, National Center for Global Health and Medicine, Tokyo, Japan.; g Alliance for Health Policy and Systems Research, World Health Organization, Geneva, Switzerland.; h Milken Institute School of Public Health, George Washington University, Washington, DC, United States of America.

The first Global Symposium on Health Systems Research was held in 2010 under the leadership of the World Health Organization and the Alliance for Health Policy and Systems Research. Since 2014, under the stewardship of Health Systems Global, the symposium has been held every two years. This biennial event has long served a key global milestone for those committed to strengthening and promoting health systems, with a firm commitment to equity. The eighth Global Symposium on Health Systems Research will be held in Nagasaki, Japan, between 18 and 22 November 2024. Over 1500 people from more than 100 countries are expected to gather to share knowledge and discuss the most pressing issues and recent developments in the science of health policy and systems. Health policy and systems research is now a well-recognized field, and the symposium will provide an opportunity to understand how this field can have a greater impact on addressing the world’s health challenges. Health Systems Global is currently accepting abstracts for proposed individual sessions and capacity-strengthening sessions.^1^

The symposium seeks to focus on key current health challenges, with the theme *Building just and sustainable health systems: centring people and protecting the planet*.^2^ Since the Declaration of Alma-Ata on primary health care in 1978,^3^ a variety of health-related initiatives have emphasized the importance of human-centredness as a principle guiding health policy and health systems. The commitment to leaving no one behind in the sustainable development goals, and the target of universal health coverage^4^^,^^5^ show that the objective to achieve health for all endures. Yet, the coronavirus disease 2019 (COVID-19) pandemic revealed the many vulnerabilities and inequities that continue to characterize health systems globally. These inequities are now intersecting with the health impacts of the climate crisis. Although awareness of the role that health systems must play in mitigating and adapting to the impacts of climate change is growing, the global health sector is struggling to make the necessary changes. Moreover, the sector itself needs to decarbonize. If it were a country, the sector would rank as the fifth greatest carbon dioxide emitter.^6^ Thus, paying attention to and promoting research on just and sustainable health systems is needed.

The symposium has four subthemes. First, strengthening health systems for planetary health emphasizes the need for research, policy and programming to ensure health systems are both climate-resilient and environmentally sustainable. The health of human and animal populations is deeply entwined. Therefore, research and policy development are needed to enable health systems to adapt to and address the complex challenges posed by climate change, urbanization, biodiversity loss, emerging diseases and pandemics.^7^^,^^8^

Second, advancing justice, inclusion and belonging in health systems calls for greater knowledge on how to build health systems that ensure everyone’s right to access health care, even when countries are in conflict or have experienced a disaster. 

Third, the subtheme of health governance, policy and institutional frameworks for just and sustainable health systems aims to address gaps in understanding how to define, design and enact effective health policy, governance and institutional frameworks for health systems. 

Fourth, the subtheme of knowledge for just health systems recognizes that the production and use of knowledge for health policy and systems is influenced by ethical, epistemological and methodological choices that in turn reflect the underlying values of practitioners, policy-makers and researchers.

We welcome all stakeholders to join us at the 2024 symposium to share, debate, connect, coordinate and collaborate – either in person or online.
